# Appyters: Turning Jupyter Notebooks into data-driven web apps

**DOI:** 10.1016/j.patter.2021.100213

**Published:** 2021-03-04

**Authors:** Daniel J.B. Clarke, Minji Jeon, Daniel J. Stein, Nicole Moiseyev, Eryk Kropiwnicki, Charles Dai, Zhuorui Xie, Megan L. Wojciechowicz, Skylar Litz, Jason Hom, John Erol Evangelista, Lucas Goldman, Serena Zhang, Christine Yoon, Tahmid Ahamed, Samantha Bhuiyan, Minxuan Cheng, Julie Karam, Kathleen M. Jagodnik, Ingrid Shu, Alexander Lachmann, Sam Ayling, Sherry L. Jenkins, Avi Ma'ayan

**Affiliations:** 1 Department of Pharmacological Sciences, Mount Sinai Center for Bioinformatics, Icahn School of Medicine at Mount Sinai, One Gustave L. Levy Place, Box 1603, New York, NY 10029, USA; 2 Pencil Worx Design, 345 West 88th Street, New York, NY 10024, USA

**Keywords:** workflow, notebooks, big data, data analysis, data visualization, RNA-seq, scRNA-seq, machine learning, TCGA, gene set enrichment analysis

## Abstract

Jupyter Notebooks have transformed the communication of data analysis pipelines by facilitating a modular structure that brings together code, markdown text, and interactive visualizations. Here, we extended Jupyter Notebooks to broaden their accessibility with Appyters. Appyters turn Jupyter Notebooks into fully functional standalone web-based bioinformatics applications. Appyters present to users an entry form enabling them to upload their data and set various parameters for a multitude of data analysis workflows. Once the form is filled, the Appyter executes the corresponding notebook in the cloud, producing the output without requiring the user to interact directly with the code. Appyters were used to create many bioinformatics web-based reusable workflows, including applications to build customized machine learning pipelines, analyze omics data, and produce publishable figures. These Appyters are served in the Appyters Catalog at https://appyters.maayanlab.cloud. In summary, Appyters enable the rapid development of interactive web-based bioinformatics applications.

## Highlights

• Appyters turn Jupyter Notebooks into full-stack web-based applications
• The Appyters Catalog serves over 75 Appyters
• Appyters provide a way to parameterize and generalize Jupyter Notebooks
• Appyter reports have permanent URLs that can be shared and published

## The bigger picture

Appyters facilitate bioinformaticians to convert their Jupyter Notebook workflows into lightweight, interactive, open-source, reproducible web-based bioinformatics applications. The Appyters Catalog is a software platform that enables biomedical researchers to analyze and visualize their data in many ways. Appyters were developed to create many bioinformatics web-based reusable workflows, including applications to build customized machine learning pipelines, analyze omics data, and produce publishable figures.

## Teaser

Appyters turn Jupyter Notebooks into fully functional standalone web-based bioinformatics applications. Appyters were used to create many bioinformatics web-based reusable workflows, including applications to build customized machine learning pipelines, analyze omics data, and produce publishable figures. These Appyters are served in an Appyters Catalog. In summary, Appyters enable the rapid development of lightweight, interactive, open-source, reproducible web-based bioinformatics applications.

## Introduction

**Figure 1:**
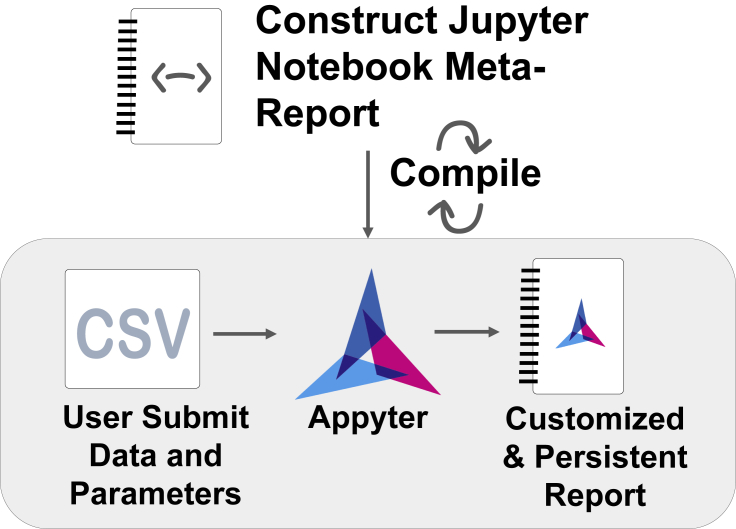
Appyters are created from a meta-Jupyter Notebook report that contains magics Once compiled, these meta-reports are converted into web-based applications that can accept user input. Once the users upload their data, and enter their parameters in a form, the notebook is automatically executed in the cloud and produces a permanent report.

Jupyter Notebooks have seen widespread adoption in data science with over 2.5 million notebooks posted on GitHub as of September 2018.^1^ With the ability to construct and view code, figures, and markdown text all in one place, Jupyter Notebooks are ideal for constructing well-documented, reproducible data analysis pipelines, promoting transparency and reusability. Because of this transparency, several web-based applications have adopted Jupyter Notebooks as a way of presenting scientific results of multistage user-configurable data analysis pipelines. For example, we developed BioJupies,^2^ an automated RNA-sequencing (RNA-seq) data analysis pipeline that enables users to upload their fastq files, or data tables, from RNA-seq gene expression profiling, to automatically receive a detailed analysis report of their data delivered as a Jupyter Notebook. Another example is Single Cell Explorer, a single-cell RNA-seq (scRNA-seq) data analysis environment.^3^ NGLview is a Jupyter widget developed to view molecular structures inside Jupyter Notebooks.^4^ These and other related tools ensure reproducibility and transparency by producing reports that can be modified both before and after execution while detailing the steps taken for each part of the analysis. Some prior efforts have been made to present Jupyter Notebooks as web-based applications for the purpose of interactive dashboards constructed directly from Jupyter Notebooks. These efforts include nbinteract,^5^ bokeh,^6^ and voila,^7^ among others. Other efforts have been made, specifically by cloud computing providers, to permit Jupyter Notebook rendering and execution directly in the web browser, or on a remote server. These include, for example, Google Colab^8^ and MyBinder.^9^ Papermill^10^ facilitates the sequential execution of cells within a Jupyter Notebook, while Bacatá^11^ provides a framework for generating notebook interfaces for domain-specific languages. XML2Jupyter^12^ dynamically generates Jupyter widgets from XML descriptions. None of these previous works facilitate low-barrier entry to Jupyter Notebook rendering and execution from a skeleton template. Such implementation can support the modification of the data or methods implemented in the notebook without requiring direct code manipulation. To accomplish this, we developed Appyters. Appyters are designed to provide experimental biologists without coding skills an easy way to execute bioinformatics workflows to analyze their data. For computational biologists, Appyters provide a framework to quickly construct and deploy bioinformatics web-based applications from their Jupyter Notebooks. One part of Appyters is the expansion of the Jupyter Notebook language to support external environment variables. Appyters can be considered a meta-Jupyter Notebook language that is compatible with standard Jupyter Notebook execution. The Appyter meta-Jupyter Notebook language adds Jupyter "magics," which permit constructing output Python code with jinja2,^13^ declaration of injectable variables by means of pre-defined or extendable "fields," and a mechanism to turn the meta-Jupyter Notebook language into a full-stack web-based bioinformatics application. Such Appyter web-based applications help users submit their data via a web-based form satisfying the defined fields followed by rendering and execution of a standard Jupyter Notebook given that input (Figure 1).

## Results

### Developing an Appyter

Appyters can be created by converting standard Jupyter Notebooks into web-based applications by inserting special code in the notebook. The Jupyter Notebook magics are set up by initializing them in the first cell of the notebook. In this way, these magics can be used to directly serialize and subsequently execute jinja2-style template syntax. This syntax permits a wide range of branches, which enable the notebook's code to be adjusted as needed based on declared “fields.” These fields represent the type of input field to be used to construct that template variable. Fields are available natively for all major data types. In addition, Fields can be extended to support more specific use cases of input form components. These fields can be extracted by inspection and are eventually used for the purpose of rendering a web-based HTML form that is the initial user interface (UI) of each Appyter. When a user of the Appyter submits the form for execution, the Appyter assembles all the necessary variables to fully serialize a customized instance of the target instantiation of the template Jupyter Notebook. Importantly, Appyter forms can accommodate a file upload input as a form field. The file upload feature facilitates uploading user-submitted files to be utilized for a given analysis.

**Figure 2:**
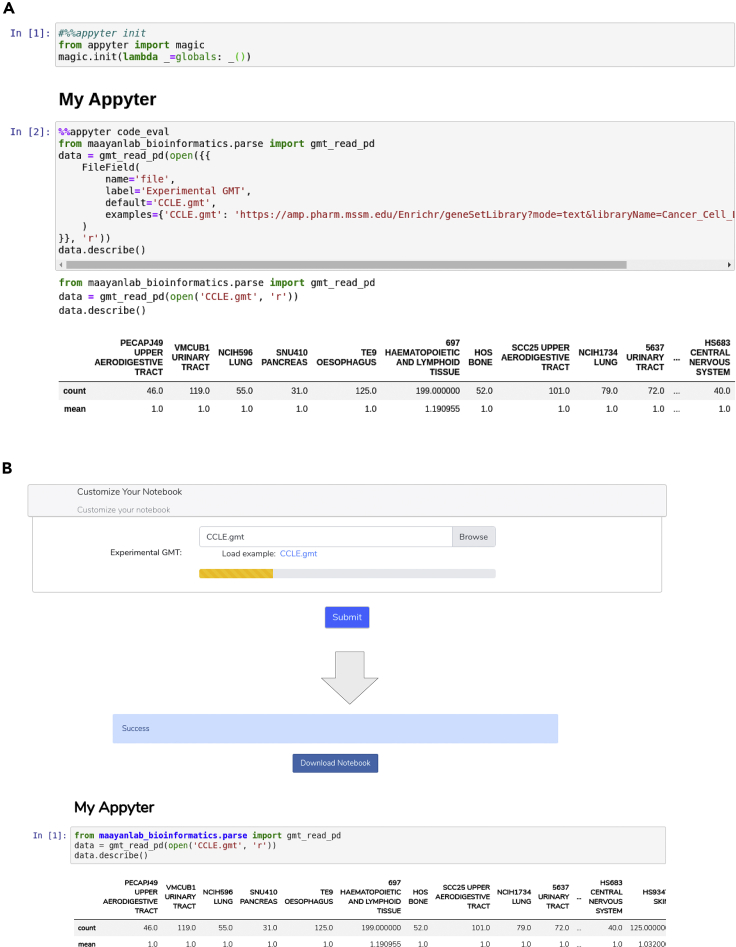
Example components when developing Appyters (A) The Appyter library provides functions to initialize Appyter-related jinja2-style template functionality using a standard Jupyter Notebook session, allowing creation and testing with default field inputs. (B) The Appyter can be served, tested, and updated in real time using the Appyter command line interface.

Appyters have several mechanisms for extension. Some of these extensions involve built-in features, and others involve overriding or extending these built-in features. “Profiles” are template pre-sets for the default application-defined fields; these enable quick beautification of the Appyter with little effort. “Extras” are feature flags that can be used to enable certain opt-in features such as adding a table of contents to the Jupyter Notebook output report or providing a button for code toggling. The Extras are independent of the Profiles. However, all existing fields and pages can be overridden or extended by means of a documented directory and a file structure. Overrides placed in the proper location are automatically loaded by the Appyter, and this makes it possible to define new fields or fine-tune the application styling without having to make modifications to the Appyter. Static files, Appyter fields, jinja2 filters, jinja2 templates, and even Flask^14^ blueprints or Dash applications can all be defined, integrated, extended, and overridden. The Appyter command line interface (CLI) can be used to interact with the Appyter feature set. This includes locating and describing available fields, profiles, and extras, as well as facilitating the inspection, construction, evaluation, and serving (via Flask) of Appyters (Figure 2). Furthermore, the CLI facilitates interacting with remote Appyter instances using both the Appyter REST application programming interface (API) and websockets. This enables the inspection, and real-time asynchronous evaluation, of public Appyter endpoints directly from the command line or as part of a workflow.

### The Appyter Catalog

**Figure 3:**
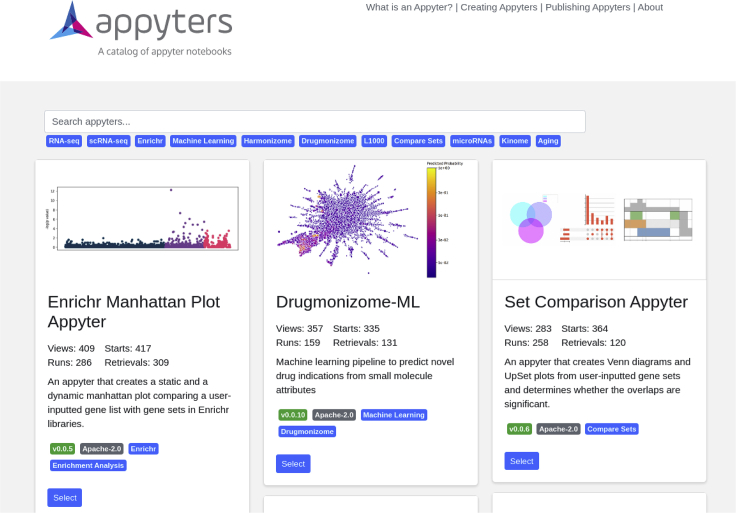
Screenshot from the Appyters Catalog with the Enrichr filter applied Each Appyter is presented as a box with tags and links. A search engine and pre-defined buttons can be used to find and filter Appyters.

The Appyter Catalog facilitates the integration of many individual Appyters into a unified web interface that presents each Appyter and provides various means of categorization and search (Figure 3). By default, Appyters are sorted by their usage statistics. Appyters can be integrated within the Appyter Catalog via GitHub pull requests. By implementing a standardized machine-validatable set of documented requirements, Appyter pull requests need to be formatted in a uniform way. These pull requests are tested automatically to ensure compliance with the Appyter guidelines to promote meaningful integration and catch obvious errors that would result in a broken production environment. A Python test suite asserts conformance of each Appyter's directory structure along with its required files. The test also triggers additional mechanisms to assert the validity of the content within those files. JSON Schema^15^ is used to validate a required Appyter JSON file that contains the name, description, versioning information, authorship, contact information, usage license, and tags for Appyter categorization purposes. Standardized requirements and Ubuntu system dependency text files are required along with the rest of the directory to construct and build a Dockerfile.^16^ The Dockerfile can run the Appyter before inspecting, constructing, and executing an instance of the Appyter with all its defaults. The individual dockerization of each Appyter simplifies the integration of the Appyter into the catalog. In this way, Appyters within the catalog can have heterogeneous requirements while enabling uniform orchestration via systems such as Marathon^17^ or Kubernetes.^18^ Relying on the structure asserted by the validation, several scripts are used to construct a Docker Compose capable of serving all the Appyters in the catalog from a single nginx^19^ ingest service. Furthermore, the Appyter Catalog is set up to enable application-wide manipulations such as versioning or HTML template overrides. This makes it possible to expand the capabilities of the underlying Appyter as well as unifying the theme across new and existing Appyters.

**Figure 4:**
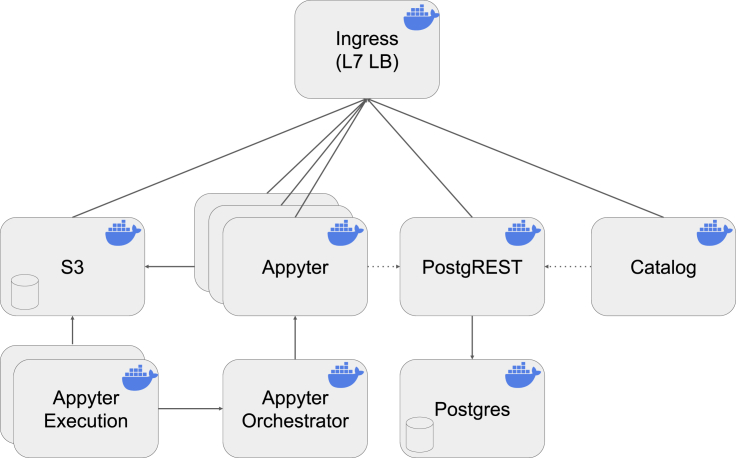
The various components constituting the Appyters Catalog system Once a user selects an Appyter to execute from the catalog, the job is counted and then enters a queue. The Appyter orchestrator then executes Appyters with Appyter data from S3. L7 LB, layer 7 load balancing.

The Appyter JSON files are aggregated and supplemented with additional information, including a README file and version information derived from the Git history. This information is made available to the frontend, which constructs the UI directly from this data model. The entire Appyter system is made of independent microservices that communicate to constitute the Appyter Catalog orchestration platform (Figure 4). The system implements a layer 7 load balancer that is used to serve all independent components onto a unified system. A cloud-agnostic S3-compatible minio is used for object storage for Appyter data. The UI is built with svelte^20^ and Bootstrap^21^ and served with nginx, while data-persistent capabilities such as page hits are deferred to a PostgreSQL database^22^ that can be accessed via a PostgREST microservice API. Thus, the frontend is a statically constructed single-page web application. Navigation to an Appyter leaves the domain of this web application handled by the accessed Appyter application. The Appyters communicate internally with the Appyter orchestrator to dispatch execution jobs on demand. Execution jobs access data directly from S3 and send updates to the Appyter web application. All the Appyter microservices are available as subcommands in the Appyter library, while the Appyter Catalog facilitates construction of Docker Compose or Kubernetes helm charts for multi-Appyter deployment.

### Appyter adherence to the FAIR guidelines

The findable, accessible, interoperable, and reusable (FAIR) guiding principles^23^ were considered while designing the Appyter system. We sought to satisfy several universal FAIR metrics as prescribed in the exemplar metrics for FAIRness.^24^ The Appyter Catalog itself promotes findability of the Appyters by enforcing that all Appyters are formatted and annotated in a uniform way. The “appyter.json” file is JSON-schema validatable. This ensures that Appyters contain a unique title, short description, spdx-compatible license identifier, authorship, and contact information. Furthermore, a semver (https://semver.org/), which must be updated to modify an existing Appyter, is present and ties the Appyter to the Docker image container, ensuring historical provenance. In addition, the entire catalog is maintained under Git version control. Results of an Appyter execution are tied to a unique sha1 checksum that is formed based on the information that went into it, resulting in reproducible globally unique identifiers. Although Appyters are not yet registered with an independent identifier provider, integration with the digital object identifier (DOI) system is planned. The instance executions can be tied directly back to the precise data and version of the executed Appyter. The Appyter concept itself promotes interoperability and reusability by ensuring that all Appyters can expose a uniform programmatic interface accessible both remotely via its REST API and locally via a Docker instance or native execution of the Appyter CLI. Thus, Appyters may quickly and easily interoperate with other web-based applications and become components of data analysis workflows. Furthermore, all Appyters are open source, and this makes the information they produce transparent and reusable.

### The execution framework

The Appyter framework has several steps of execution derived from a single Jupyter Notebook, each working in tandem. Each step of the execution is available via the Appyter CLI. The Jupyter nbinspect method extracts out the fields described in the Appyter JSON format detailing those fields. This JSON represents the parameters available to construct the Appyter. The Jupyter nbconstruct method takes a JSON of values reflecting available parameters and serializes it into the complete Jupyter Notebook. The Jupyter nbexecute method then executes the Jupyter Notebook cell by cell while reporting progress as the notebook is executed. In this way, each Appyter has everything needed to run the application as a production webserver either natively or within a Docker container. Using supervisord, a process control system, the Appyter renders the form directly from the Jupyter Notebook. The complete application is served on a UNIX socket behind an nginx load balancer with supervisord. The load balancer also handles serving static files that are part of the Appyter. These files are directly accessed from the user data storage. The Appyter webserver uses aiohttp, an asynchronous-io framework, with socketio-provided websockets for real-time message passing between a user and the backend. The websocket is used to transmit files during upload and real-time cell updates during notebook execution. The webserver also serves several REST APIs that are responsible for passing form uploads through nbconstruct.

To achieve cloud-agnostic scalability, Appyters have built-in mechanisms to operate in a multi-Appyter multiexecution setting. To achieve this in a way that is independent of the underlying orchestration platform, the Appyter system has a submodule orchestration with extension modes that permit the set of Appyters to run together by different mechanisms (Figure 4). Specifically, the orchestration module consists of three main parts: the orchestrator, the dispatchers, and the jobs. The orchestrator is an internal REST API that queues execution requests and translates them into a platform-specific dispatch. Currently, three dispatchers are supported, but more could be added: “native dispatch” runs a job using an independent Appyter process, “docker dispatch” triggers the job using Docker, and “kubernetes dispatch” triggers the job using the Kubernetes API. The “job” is responsible for running nbexecute and reporting progress in real time, which it does by connecting back to the socketio room that the user triggered. Several failure modes are considered and handled both in the job and on the client side. This ensures that edge cases, including network disconnections, new users joining/leaving the room midexecution, and other scenarios, are resolved as efficiently and completely as possible. Different environments also necessitate additional requirements. Although Docker solves many issues related to per-Appyter dependency management, networked file system backends such as S3 are necessary when dealing with environments that may execute jobs on different systems. This is achieved by abstracting the file system access used throughout the application. Currently, Appyters support both the native file system and S3-compatible modes of file access. The Docker images enable the user to run a given Appyter from the CLI in an environment prepared with all the necessary dependencies.

### The initial collection of Appyters in the Appyters Catalog

So far, we have developed over 40 Appyters. These can serve as examples for the community to contribute their own Appyters to the catalog. Below, we outline short descriptions of several of the existing Appyters. For each Appyter, we describe the motivation and background for creating the Appyter and the computational tasks that can be performed with each Appyter.

#### The Bulk RNA-Seq Analysis Appyter

**Figure 5:**
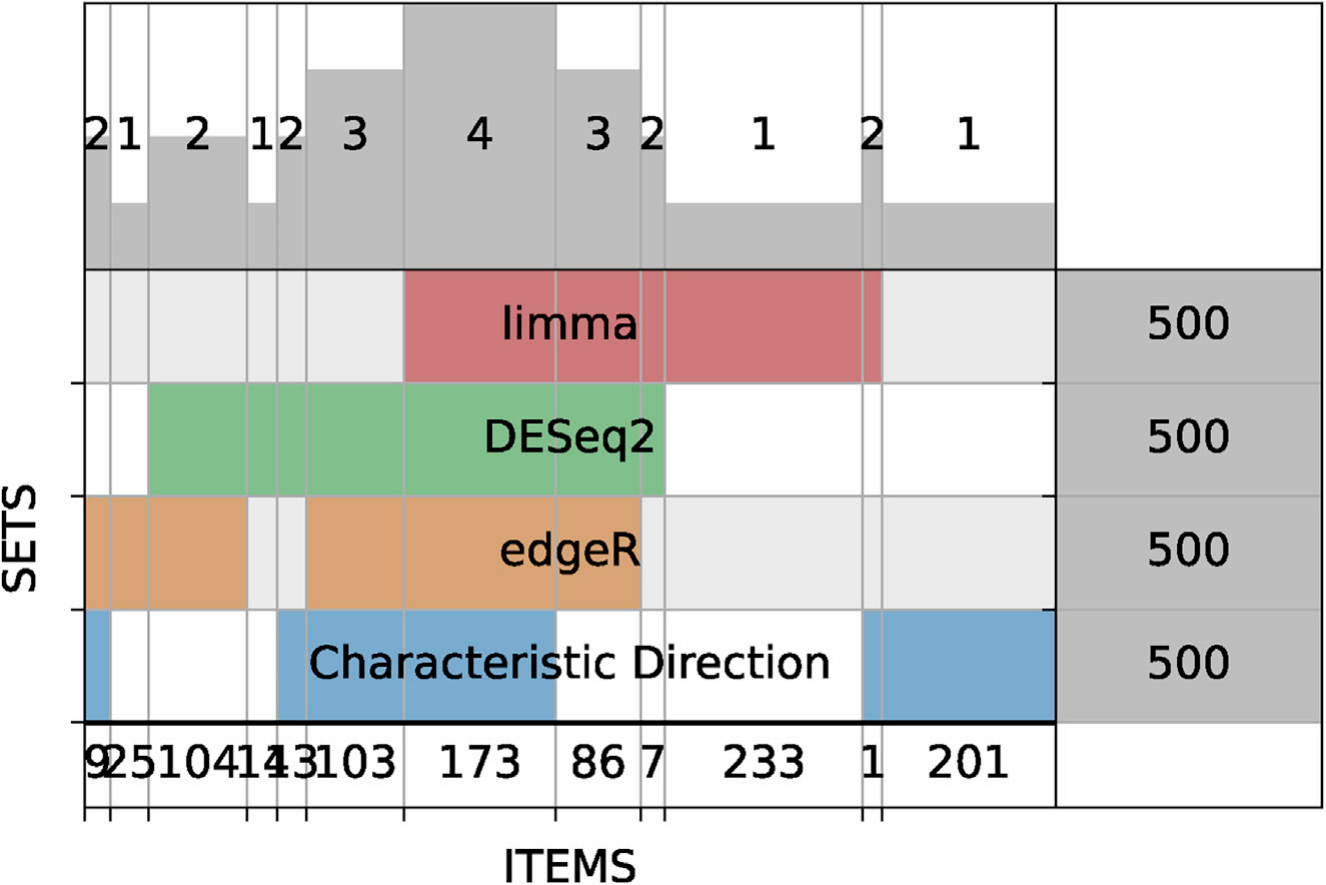
Overlap among the top-ranked 500 differentially expressed genes computed for data downloaded from the GEO study GSE70466 The differentially expressed genes are determined using the Bulk RNA-Seq Analysis Appyter, and the visualization is achieved with a SuperVenn diagram implemented within the Set Comparison Appyter.

Gene expression profiling with RNA-seq is now the most common method used to profile gene expression at the genome-wide scale.^25^ Processing RNA-seq data requires several key steps, including alignment, quantification, quality control assessment, normalization, dimensionality reduction, clustering, differential expression analysis, and pathway and network analyses. We and others developed several RNA-seq data analysis pipelines that cover many of these steps.^26^, ^27^, ^28^, ^29^, ^30^ The Bulk RNA-Seq Analysis Appyter enables non-computational users to analyze and visualize their own RNA-seq datasets with an array of downstream analysis and visualization tools. The Appyter starts with an expression matrix of raw read counts and a file that provides metadata describing each sample. First, the Appyter implements various data normalization methods such as counts per million, log transformation, *Z* score normalization, and quantile normalization, which are applied to the raw read counts. To visualize the normalized data, dimensionality reduction is implemented with principal-component analysis (PCA),^31^ t-distributed stochastic neighbor embedding (t-SNE),^32^ and uniform manifold approximation and projection (UMAP).^33^ The results from these methods are visualized as a 3D interactive scatterplot. Next, hierarchical clustering is performed with Clustergrammer,^34^ an interactive Jupyter widget that produces interactive heatmaps from gene expression data tables. Clustergrammer enables users to identify clusters of samples and modules of genes. The Appyter also produces a library size analysis that calculates and displays the total reads mapped for each RNA-seq sample. This analysis facilitates the identification of outlier samples and provides assessment of the overall quality of the data. Next, the Bulk RNA-Seq Analysis Appyter computes gene expression signatures. Several differential gene expression methods are implemented, including limma,^35^ the Characteristic Direction,^36^ edgeR,^37^ and DESeq2.^38^ The differential expression results are visualized as volcano plots and MA plots. Because the Appyter has implementations of four different methods that compute differential expression, it is relatively straightforward to compare the similarities and differences among the gene sets called by these methods. To illustrate this concept, we used the Set Comparison Appyter to visualize the intersection among the top 500 upregulated genes as determined by these four methods when applied to the Bulk RNA-Seq Analysis Appyter example. The example is taken from an unpublished study (Gene Expression Omnibus [GEO] accession GSE70466) that compared normal and cancerous prostate cell lines, LNCaP (n = 3) and PrEC (n = 3). In general, we can quickly see that about 60% of the same genes rank in the top 500 genes produced by each of the four methods (Figure 5). We do not intend to provide here an exhaustive analysis that compares these methods, but simply demonstrate that various Appyters can be used to perform such analyses with ease. Gene set enrichment analysis within the Bulk RNA-Seq Analysis Appyter is applied to the up/downregulated genes with Enrichr.^39^ The enrichment analysis results from key libraries are displayed directly within the Appyter. These libraries include Gene Ontology,^40^ KEGG,^41^ Reactome,^42^ WikiPathway,^43^ ChEA,^44^ ENCODE,^45^ KEA^46^,^47^ and miRTarBase.^48^ Finally, the computed gene expression signatures are submitted to L1000CDS2^49^ and L1000FWD,^50^ which are two web-based search engines that match input gene expression signatures with gene expression signatures generated by the Library of Integrated Network-Based Cellular Signatures (LINCS)^51^ L1000 assay.^52^ Overall, the Bulk RNA-Seq Analysis Appyter provides detailed reports that can enable experimental biologists to extract more knowledge from their RNA-seq data. The Bulk RNA-Seq Analysis Appyter is available at https://appyters.maayanlab.cloud/#/Bulk_RNA_seq.

#### The scRNA-Seq Analysis Appyter

Since the first publication of scRNA-seq in 2009,^53^ scRNA-seq profiling has been improved in cost and quality, and as a result the number of scRNA-seq studies deposited into GEO^54^ has been rapidly growing. To assist experimental biologists with no computational skills in analyzing their scRNA-seq data, and to explore published scRNA-seq data, we developed the scRNA-Seq Analysis Appyter. First, the Appyter evaluates the quality of the scRNA-seq data by examining the expression level of mitochondrial genes. High expression level of mitochondrial genes indicates poor scRNA-seq data quality.^55^, ^56^ This is because when the cell membrane is lysed, cytoplasmic RNA is lost, but mRNA enclosed in the mitochondria is retained. This analysis removes single cells that likely had lysed cell membranes during mRNA extraction. Next, library size analysis is applied to each sample, and a bar chart is produced to visualize the total reads mapped to each sample. This analysis helps identify outlier samples and assess the overall quality of the scRNA-seq data. Next, the scRNA-Seq Analysis Appyter performs several standard data normalization methods developed specifically for scRNA-seq data analysis.^57^, ^58^, ^59^ These methods convert the raw reads into standardized measures of gene expression by removing confounding factors that may affect downstream analysis. After normalization, the Appyter visualizes the most highly expressed genes across all samples. Then, dimensionality reduction is performed using PCA.^31^ Next, the scRNA-Seq Analysis Appyter performs data imputation with the Markov affinity-based graph imputation of cells (MAGIC) method.^60^ An interactive hierarchical clustering heatmap is generated with Clustergrammer^34^ to enable exploring similarity between samples and to identify co-expression gene modules. The scRNA-Seq Analysis Appyter also provides differential gene expression analysis with the same four methods implemented for the Bulk RNA-Seq Appyter, namely, limma,^35^ the Characteristic Direction,^36^ edgeR,^37^ and DESeq2.^59^ Clusters of single cells are identified with the Leiden algorithm,^61^ which automatically labels samples based on their optimal cluster membership assignment. Differential expression between clusters is then computed by comparing each cluster with the others. The differentially expressed genes for each cluster are submitted to Enrichr^39^ for enrichment analysis. Next, trajectory inference analysis, which arranges cells based on their progression through the differentiation process, is implemented with three independent methods: diffusion pseudotime,^62^ Monocle,^63^ and Tempora.^64^ Tempora is a pathway-based single-cell trajectory inference method that infers the developmental lineage of single cells based on pathway information. To predict cell types, Digital Cell Sorter^65^ is implemented to categorize single cells into their hematopoietic lineage. In the future, we plan to add additional cell type prediction algorithms. In summary, the scRNA-Seq Analysis Appyter provides an example scRNA-seq analysis workflow that can be used by experimental biologists to glean knowledge about their scRNA-seq data by uploading their data into an online form. The scRNA-Seq Analysis Appyter is available at https://appyters.maayanlab.cloud/#/scRNA_seq.

#### The Harmonizome-ML Appyter

The Harmonizome resource^66^ provides a collection of processed datasets gathered to serve and mine knowledge about genes and proteins from >100 major online resources and repositories. To create the Harmonizome, we extracted, abstracted, and organized data into >100 million functional associations between human genes and their attributes. Such attributes can be physical interactions with other biomolecules, expression in cell lines and tissues, genetic associations with human phenotypes, or changes in expression after drug treatment. We stored these associations in a relational database along with rich metadata for the genes, their attributes, and the original resources. The Harmonizome-ML Appyter provides on-the-fly imputation of knowledge about genes and proteins with machine learning using data from the Harmonizome resource.^66^ By integrating knowledge from a variety of high-content omics resources with low-content literature-based knowledge, it is possible to predict gene and protein function with machine learning. The Harmonizome-ML Appyter empowers non-coding users to perform gene and protein knowledge imputation. Users can select attributes for training from a variety of processed omics datasets and predict almost any biological function for genes, such as associations with human diseases, membership in cell signaling or metabolic pathways, and knockout mouse phenotypes. Harmonizome-ML first presents the user with a form on which they can choose from a collection of processed omics datasets to use as the attributes for learning and a class of knowledge to predict. Users can then select from a variety of machine learning algorithms, their various parameter settings, and the model performance evaluation methods. Once those options are entered, the Harmonizome-ML Appyter generates a report that contains the predictions with an assessment of the predictive model performance. The output Jupyter Notebook produced by the Harmonizome-ML Appyter provides an opportunity to modify the code for customized analyses. Using Harmonizome-ML, investigators can quickly explore machine learning-backed predictions for understudied gene-gene function associations to guide their research. The Harmonizome-ML Appyter is available at https://appyters.maayanlab.cloud/#/harmonizome_ml.

#### The Drugmonizome-ML Appyter

A wealth of data from a multitude of sources for thousands of bioactive small molecules is readily available. This information could be harnessed to develop machine learning models that utilize such harmonized data to predict the properties of small molecules that are poorly annotated. The Drugmonizome database draws upon a variety of publicly available resources to label each compound by its associations with protein targets, induced gene expression profiles, chemical features, and other attributes. The Drugmonizome-ML Appyter is built on top of the Drugmonizome (https://maayanlab.cloud/drugmonizome/) datasets to predict novel indications and other attributes such as drug targets or side effects for poorly annotated bioactive small molecules with machine learning. The machine learning model constructed by the Drugmonizome-ML Appyter uses the scikit-learn package,^67^ which provides a variety of options for various canonical classification algorithms and dimensionality reduction techniques, as well as feature selection and cross-validation methods. These options can be selected from the Drugmonizome-ML Appyter form. When executed, the Drugmonizome-ML pipeline trains a model for each cross-validation split to predict properties for all available drugs and small molecules. The cross-validated model performance is displayed with receiver operating characteristic and precision-recall curves. The results from such analysis can be used to assign novel indications for existing drugs and other small molecules. When recursive feature selection is selected, the relative importance of individual input features for a specific prediction task is assessed. Examining feature importance improves the interpretability of Drugmonizome-ML-generated models. In addition, important features may suggest drug attributes to consider for therapeutic design, or for discovering aspects of the biology playing a role in the underlying mechanisms of action of the drug. In summary, Drugmonizome-ML is a general-purpose machine learning platform that can be used for predicting drug and small-molecule attributes using rapidly accumulating pharmacological knowledge. The Drugmonizome-ML Appyter is available at https://appyters.maayanlab.cloud/#/Drugmonizome_ML.

#### The Patient Cohorts RNA-Seq Viewer Appyter

The Patient Cohorts RNA-Seq Viewer Appyter provides a customizable interface for processing, visualizing, and analyzing RNA-seq data from patient cohorts. The goal of the Appyter is to provide comprehensive analysis of patient cohorts by considering the RNA-seq profiling of the patient samples together with information about their clinical parameters. The Appyter automatically identifies clusters of patients based on their RNA-seq profiles and associates clinical metadata with each cluster. The Appyter is preloaded with example data collected by The Cancer Genome Atlas (TCGA)^68^ but can accommodate other user-uploaded patient cohort RNA-seq datasets. A standard RNA-seq pre-processing pipeline, including normalization and dimensionality reduction, is implemented. Clusters of patients and their associated differential gene expression profiles are fed into a series of downstream analyses. These include survival analysis with Kaplan-Meier plots, enrichment analysis with Enrichr,^39^ and small-molecule and drug prioritization based on the L1000 data with L1000FWD.^50^ These analyses provide insights into each cluster's unique genomic, transcriptomic, and clinical features. In summary, the Patient Cohorts RNA-Seq Viewer Appyter enables researchers with no programming background to perform complex analyses to uncover patterns embedded in their RNA-seq patient cohort datasets. The Patient Cohorts RNA-Seq Viewer Appyter is available at https://appyters.maayanlab.cloud/#/Patient_Cohorts_RNASeq_Viewer.

#### Appyters to extract, transform, and load data for Harmonizome

The Harmonizome Extract, Transform, and Load (ETL) suite of Appyters contains pipelines to convert omics resources into a format that is compatible with the Harmonizome data model. These Appyters enable the loading of files downloaded from the various online biomedical resources that have available processed data within Harmonizome.^69^ After uploading these files, the Appyters filter, normalize, and standardize the uploaded raw data to create a Harmonizome-compatible output, which includes gene set libraries, bipartite graphs, and attribute tables. Data summaries are visualized to examine the ingested harmonized data within each Appyter. In summary, the Harmonizome ETL suite of Appyters makes it easy to maintain the Harmonizome resource by having coding-free workflows to transform data into the harmonized format provided by Harmonizome. On their own, the Harmonizome ETL Appyters in this suite serve up-to-date harmonized and abstract biomedical data from multiple key resources useful for many other applications. There are currently 38 Harmonizome ETL Appyters, which are available from https://appyters.maayanlab.cloud/#/?tags=Harmonizome&q=ETL.

#### Enrichr visualization Appyters

**Figure 6:**
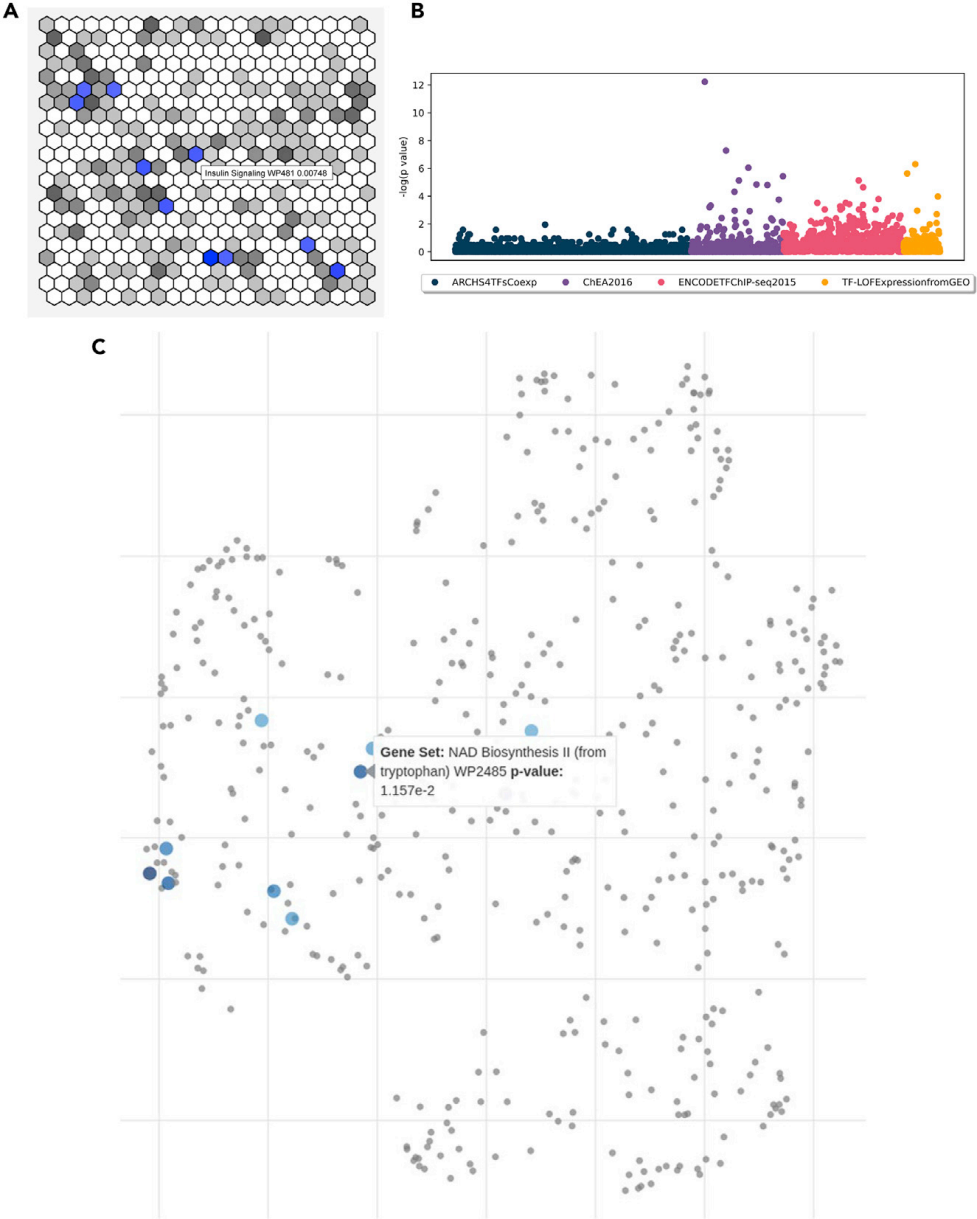
Three methods of visualization of gene set enrichment analysis results (A) Hexagonal grid visualization places all the terms from a gene set library near one another based on gene set content similarity. Top enriched terms are highlighted in blue. (B) Manhattan plot visualization of enrichment results for four gene set libraries from Enrichr. (C) Scatterplot visualization of enrichment results. Each point represents a gene set. The points are scattered based on their gene set similarity. Points highlighted in blue are enriched terms. For all analyses default settings and example files were used.

Gene set enrichment analysis is a computational method that enables the identification of underlying biological functions and processes for a given experimentally determined input gene set. Enrichment analysis results are commonly communicated in publications as tables and bar charts. However, bar charts can accommodate the visualization of only a small subset of the enriched terms and do not convey the similarity among enriched gene sets. Utilizing the Enrichr API,^39^ three Appyters generate alternative visualizations for enrichment analysis results. The Canvas Enrichment Analysis Appyter creates hexagonal grids in which each hexagon represents a gene set from an Enrichr library. The hexagons are arranged so that similar gene sets are grouped together, and this is achieved via offline simulated annealing of each library. Hexagons are colored with varying intensity depending on the enrichment analysis p values, with most hexagons colored in gray, while enriched terms are colored in purple (Figure 6A). The Manhattan Plot Enrichment Analysis Appyter creates both static and dynamic Manhattan plots to visualize enrichment analysis p values for multiple libraries at once (Figure 6B). The Scatter Plot Enrichment Analysis Appyter creates scatterplot visualizations of each Enrichr gene set library wherein each point represents a gene set from the library and similar gene sets are clustered together (Figure 6C). This arrangement is determined and created using offline multidimensional scaling^70^ and term frequency-inverse document frequency calculations. The points are colored blue if the terms they represent are significantly enriched compared with the user-input gene list. In summary, the collection of these three enrichment analysis data visualization Appyters provides researchers with alternative methods to visualize their gene set enrichment analysis results. The Enrichr Visualization Appyters are available at https://appyters.maayanlab.cloud/#/?tags=Enrichr&q=skylar.

#### Set Comparison Appyter

Although there are bioinformatics tools developed to compare gene sets, including creating Venn diagrams from several input gene sets, important features such as p value overlap calculation and links to downstream analyses tools are commonly missing from current applications. The Gene Set Comparison Appyter can be used to generate a complete report that compares two to six gene sets. The Appyter generates Venn, UpSet, and SuperVenn diagrams from the input gene sets. To further analyze overlapping sets, set intersections are linked to downstream enrichment analysis with Enrichr.^39^ Moreover, the Fisher exact test is calculated to determine whether the overlap between input gene sets is significant. This test is performed on all possible pairs of sets. The results of these tests are displayed in a table as a heatmap. The users of the Set Comparison Appyter can customize the color of the display items and save the figures in multiple formats. In summary, the Gene Set Comparison Appyter is a useful tool for experimental and computational biologists to compare their gene sets and generate publication-ready graphics. The Set Comparison Appyter is available at https://appyters.maayanlab.cloud/#/CompareSets.

#### Other Appyters

There are several additional Appyters that are not described here in detail. For example, there are numerous Appyters that perform ETL to process data describing properties of drugs and small molecules. There are also Appyters to perform kinase enrichment analysis,^47^ predict gene function for non-coding genes using RNA-seq co-expression data, perform cross-linking immunoprecipitation-seq analysis, analyze the LINCS L1000 shRNA knockdown data,^52^ and analyze the LINCS KinomeScan data.^51^ A selected list of 27 Appyters that are currently provided within the Appyter Catalog is provided (Table 1).

**Table 1:** List of Appyters currently available from the Appyter Catalog

Appyter name	URL to Appyter (from base URL^a^)	Description
Bulk RNA-Seq	Bulk_RNA_seq	generates reports for bulk RNA-seq downstream analysis
ChIP-Seq	ChIP_seq	generates reports for downstream ChIP-seq analysis
Compare Sets	CompareSets	compares sets with Venn diagrams and UpSet plots
DrugShot	DrugShot	converts PubMed search terms to drug sets based on co-occurrence
Drugmonizome Consensus Terms	Drugmonizome_Consensus_Terms	combines drug set enrichment analysis for multiple drug sets
Drugmonizome ML	Drugmonizome_ML	produces machine learning pipelines for predicting drug properties
Enrichr Visualizer	Enrichment_Analysis_Visualizer	produces visualization of Enrichr enrichment results for one library
Enrichr Canvas	Enrichr_Canvas_Appyter	visualizes Enrichr results as hexagonal canvas for multiple libraries
Enrichr Consensus Term	Enrichr_Consensus_Terms	combines Enrichr enrichment analysis results for multiple gene sets
Enrichr Manhattan Plot	Enrichr_Manhattan_Plot	visualizes Enrichr results as a Manhattan plot
Enrichr Scatterplot	Enrichr_Scatterplot_Appyter	visualizes Enrichr results as a scatterplot
Enrichr Compressed Bar Chart	Enrichr_compressed_bar_chart_figure	visualizes Enrichr results as a bar chart
GTEx Tissue RNA-Seq Analysis	GTEx_Tissue_RNA_Analysis	creates notebooks for human tissues profiled with RNA-seq
Gene Aging Trends	Gene_Age_Trends_Appyter	displays changes in expression for genes at different ages
Gene Conversion	Gene_Conversion_Appyter	converts tables of gene expression data from GEO to Entrez genes
ncRNAs Gene Function Predictions	Gene_Level_Functional_Predictions	predicts gene function for non-coding gene based on co-expression
Kinase Enrichment Analysis	KEA3_Appyter	performs kinase enrichment analysis to associate kinases with proteins
KINOMEscan Data Visualization	KINOMEscan	associates small molecules with kinases and kinases with small molecules
L1000FWD Consensus Drugs	L1000FWD_Consensus_Drugs	combines L1000FWD queries to rank consensus drugs and small molecules
L1000 Knockdown Search	L1000KD2	queries the LINCS L1000 shRNA knockdown dataset
Patient Cohorts RNA-Seq Viewer	Patient_Cohorts_RNASeq_Viewer	generates reports from RNA-seq data collected from patient cohorts
PrismEXP	PrismEXP	predicts gene function based on vertical partitioning of co-expression data
TCGA Data Loader	TCGA_Data_Loader	converts TCGA data into data frames for easy load into workflows
Example Appyter	Example	provides a simple Appyter to demonstrate how an Appyter works
Harmonizome ML	harmonizome_ml	produces machine learning pipelines for predicting gene properties
CLIP-Seq miRNA Analysis	miRNA_Target_Discovery	generates reports for bulk CLIP-seq downstream analysis

a The base URL for accessing these Appyter is https://appyters.maayanlab.cloud/. The base URL for accessing the source code is https://github.com/MaayanLab/appyter-catalog/tree/master/appyters.

## Discussion

It is expected that the collection of Appyters will continually grow by contributions from the community. This is because developing such web-based bioinformatics applications requires much less effort and skill compared with other alternatives. One such alternative is developing bioinformatics applications with R Shiny,^71^ a framework to convert R code into web-based applications. Many bioinformatics tools and web-based resources are developed with R Shiny. Appyters are different from R Shiny in many ways, but the most central difference is that Appyters convert a Jupyter Notebook into a web app, while R Shiny applications require the developer to write the server and client components of the application. The R environment also has notebooks called R markdown.^72^ Like Jupyter Notebooks, R markdown notebooks contain code, markdown text, and generated output such as static and interactive figures, but currently there are no simple ways to convert R markdown notebooks to web-based applications. It should be noted that Appyters can support R code in notebooks. Another emerging notebook technology is Observable.^73^ Observable is leveraging the flexible and modular capabilities of the JavaScript library D3^74^ to create interactive web-based notebooks. It is undetermined yet how this technology will influence the implementation of bioinformatics applications. One of the challenges with serving executable computational pipelines in the cloud is managing cloud costs and server resources. The Appyter Catalog currently can support concurrent users who execute several Appyters via a queuing system, but scaling can become challenging if more users utilize the system. To manage costs, we have currently set a global execution cap and share execution costs among all users. To achieve scalable management of resources it may be required to add user accounts and require heavy users to share the expense of executing their Appyters. Alternatively, the Appyters can be deployed on cloud resources such as Google Colaboratory,^8^ Kaggle (https://www.kaggle.com/), and Binder,^75^ or other platforms that provide similar services. Currently, Appyters do not offer a way to directly interact with the notebook while it is running. Google Colaboratory,^8^ Kaggle (https://www.kaggle.com/), and Binder offer interactive execution of Jupyter Notebooks in the cloud but with similar execution limitations. To enable such a feature users will be required to log in to perform Appyter execution. It is expected that more systems that enable execution of bioinformatics workflows in the cloud will become available in the coming years. This will require users to establish accounts on these systems and then export their Appyters into these accounts. In most cases, users will be able to follow the simple instructions we provide to deploy their Appyters locally, or at any remote machine. We also plan to enable user accounts on the Appyter Catalog. Such user accounts will enable users to control the privacy of their Appyters. Private accounts will also enable users to store their data together with their analysis pipelines and their results in the cloud. Although Appyters offer a way to rapidly convert Jupyter Notebooks into fully functioning web applications, not all Jupyter Notebooks are suitable for conversion into Appyters, and not all web-based bioinformatics applications can be converted into Appyters. Appyters provide a way to parameterize and generalize a Jupyter Notebook for constructing a template data analysis workflow. Details such as the incoming file format, or data cleaning and normalization steps, are often specific to each instance of a workflow. If these assumptions are not met when a user uploads his or her input file, the user may face execution errors or incorrect output. To mitigate this issue, the burden is placed on the Appyter developer to clarify important assumptions up-front, provide appropriate options, and develop validation functions that provide feedback to the user. The rapid expansion of biotechnologies that produce different types of biological data is continually increasing in variety and volume. Open and freely available bioinformatics software that properly extracts knowledge from such data is always a few years behind. Currently, non-coding users who collect data using various advanced biotechnologies must resort to establishing collaborations with computational biologists to analyze their data because robust tools do not exist yet. Appyters potentially provide an opportunity to close this gap because the framework can assist data scientists with publishing their workflows in a way that enables non-coding users to rerun those workflows on their own data. In addition, in many cases, non-coding users are not aware of the details of the computational workflows applied to their data. With Appyters, there is a permanent record that they can obtain and publish. Appyters can also become micro-publications where the Appyter itself, or its instantiations, can become citable. Furthermore, Appyters can potentially become embedded within other Appyters to construct workflows from building block Appyters. Finally, while our initial Appyter applications are all focused on bioinformatics workflow implementations, Appyters enable agile development of apps across many other scientific and non-scientific fields.

## Experimental procedures

### Resource availability

#### Lead contact

Further information and requests for digital resources should be directed to and will be fulfilled by the lead contact, Avi Ma’ayan (avi.maayan@mssm.edu).

#### Materials availability

This study did not generate new unique reagents.

#### Data and code availability

The Appyter Catalog and documentation are available at https://appyters.maayanlab.cloud/.

The code for Appyters is available on GitHub at https://github.com/MaayanLab/appyter.

The code for the Appyters Catalog is available on GitHub at https://github.com/MaayanLab/appyter-catalog.
